# Duration and Predictors of “In‐Need” Contraceptive Discontinuation in Ethiopia: A Competing Risks Analysis Using the Fine–Gray Subdistribution Hazards Model

**DOI:** 10.1155/bmri/9045082

**Published:** 2026-06-16

**Authors:** Asfawosen Aregay Berhe, Abraham Aregay Desta, Desalegn Meresa, Ataklti Gebretsadik Woldegebriel, Nega Mamo Bezabih, Gebreslassie Alemseged Bezabih, Hayelom Kahsay, Samson Atsbeha Tafere, Brhane Ayele

**Affiliations:** ^1^ Laboratory Research, Diagnostic and Quality Assurance Directorate, Tigray Health Research Institute, Mekelle, Tigray, Ethiopia; ^2^ Public Health Research and Emergency Management Directorate, Tigray Health Research Institute, Mekelle, Tigray, Ethiopia; ^3^ College of Health Sciences, Mekelle University, Mekelle, Tigray, Ethiopia, mu.edu.et; ^4^ Office of the General Director, Tigray Health Research Institute, Mekelle, Tigray, Ethiopia

**Keywords:** competing risks, contraceptive discontinuation, EDHS, Ethiopia, Fine–Gray subdistribution hazards model, in-need discontinuation

## Abstract

**Background:**

Contraceptive discontinuation while a need for pregnancy prevention persists, often termed “in‐need” discontinuation, remains a significant barrier to achieving reproductive health goals in Ethiopia. Traditional survival analyses often overestimate this risk by failing to account for competing events, such as discontinuation due to an intentional desire for pregnancy.

**Methods:**

This study utilized nationally representative data from the 2016 Ethiopian Demographic and Health Survey (EDHS). A total of 1617 contraceptive episodes among currently married women were analyzed. To identify predictors of “in‐need” discontinuation while strictly accounting for the competing risk of “desire for pregnancy,” a Fine–Gray subdistribution hazards model was employed as the primary inferential framework.

**Results:**

The overall 12‐month–adjusted cumulative incidence of “in‐need” discontinuation was heterogeneous across sociodemographic strata. Women in the 35–49 age cohort exhibited a significantly higher subdistribution hazard (2.91%) compared with those aged 15–24 (1.58%; aSHR: 1.85; 95% CI: 1.26–2.71). Secondary education was protective, reducing the 12‐month incidence to 1.27% compared with 1.85% for those with no formal education (aSHR: 0.62; 95% CI: 0.40–0.96). Conversely, women engaged in manual labor faced an increased probability of discontinuation (1.97%) compared with professional workers (1.29%; aSHR: 1.56; 95% CI: 1.08–2.25). Notably, women with undecided fertility preferences showed the highest persistence, with a 12‐month probability of only 1.03% (aSHR: 0.55; 95% CI: 0.32–0.93). Religious affiliation remained a strong predictor, with Catholic users exhibiting a higher subdistribution hazard (aSHR: 4.65; 95% CI: 2.06–10.49).

**Conclusion:**

“In‐need” discontinuation in Ethiopia is driven by a complex interplay of age, educational attainment, and occupational physical demands. The finding that older and less‐educated women are at higher risk suggests that national family planning programs should move beyond adolescent‐focused strategies to include targeted clinical counseling for long‐term users. Furthermore, the economic impact of side effects on manual laborers necessitates more flexible, community‐based follow‐up care to ensure contraceptive security for Ethiopia′s most economically vulnerable women.

## 1. Introduction

Contraceptive use is globally recognized as a fundamental public health intervention, pivotal for achieving the Sustainable Development Goals (SDGs) by reducing high‐risk pregnancies and lowering maternal and child mortality [1–3]. In developing countries, where over three‐quarters of women have utilized contraception at some point in their lives, the success of family planning programs is measured not merely by initial uptake but by the sustained continuation of use [4, 5].

However, contraceptive discontinuation—the cessation of a method while a woman is still at risk of unintended pregnancy—remains a critical challenge. Global data indicate that 12‐month discontinuation rates for reversible methods frequently range from 20% to 50% [6]. In Ethiopia, where the maternal mortality ratio remains high at 412 deaths per 100,000 live births, unintended pregnancies resulting from premature discontinuation account for approximately 24% of pregnancies among young women [7–8].

A significant limitation in existing reproductive health literature is the inconsistent definition of the “discontinuation” event. Traditional studies often aggregate all reasons for stopping, including the intentional desire for pregnancy. This overestimates the “failure” of family planning programs by failing to distinguish between women who have achieved their reproductive goals and those who stop “in‐need” due to side effects, health concerns, cost, or lack of social support [6, 9]. This study specifically addresses this gap by isolating “In‐Need Discontinuation” as the primary outcome. By treating the desire for pregnancy as a competing risk rather than a simple failure, this research provides a more precise estimation of the barriers to contraceptive persistence [10].

Evidence from sub‐Saharan Africa suggests that the hazard of discontinuation is influenced by a complex interplay of socioeconomic factors, educational attainment, and the quality of clinical counseling [11–13]. In Ethiopia, although previous regional studies have explored these factors within specific health‐facility settings [14], their findings often lack generalizability to the broader, community‐based population due to localized sociocultural and service‐delivery variations [15–18]. Furthermore, few national studies have utilized an appropriate statistical modeling framework to handle the time‐dependent nature of these risks under competing environments. The hazard of discontinuation is rarely constant; it changes dynamically as users navigate the initial months of method adoption and transition into long‐term maintenance [19].

To address these critical evidence gaps, this study utilizes the 2016 Ethiopia Demographic and Health Survey (EDHS) contraceptive calendar to employ a retrospective cohort design. By applying a Fine–Gray subdistribution hazards model, we aim to evaluate the duration and predictors of “in‐need” discontinuation among a nationally representative sample of married women. This approach accounts directly for the desire for pregnancy as a competing event, providing more accurate parameters for public health planning.

## 2. Materials and Methods

### 2.1. Study Setting and Data Source

This study employed a retrospective cohort design using secondary data from the 2016 EDHS [20]. Ethiopia, the second most populous nation in Africa, is characterized by significant sociodemographic diversity, with a labor market heavily reliant on agriculture (46.3% of GDP) [20, 21]. Data were extracted from the Woman′s Questionnaire contraceptive calendar, which retrospectively captured a 60‐month history of contraceptive use, including initiation dates, termination dates, and specific reasons for discontinuation [20, 21].

### 2.2. Study Population and Sampling

The study population consisted of women of reproductive age (15–49) in Ethiopia who utilized modern contraception within the 5 years prior to the survey. The final analytical sample was restricted to 2318 contraceptive episodes among currently married women. To maintain a homogeneous risk group, we excluded episodes involving sterilization, as well as women who were never married, divorced, separated, or widowed at the time of the survey [20].

### 2.3. Variable Measurements and Operational Definitions

#### 2.3.1. Unit of Analysis

The primary unit of analysis was the “contraceptive episode,” defined as a period of continuous use of a specific modern method.

#### 2.3.2. Primary Outcome (In‐Need Discontinuation)

The outcome was strictly defined as “In‐Need Discontinuation.” This refers to the cessation of a method due to side effects, health concerns, husband′s opposition, or cost, among women who still desired to prevent or delay pregnancy.

#### 2.3.3. Competing Risks

Observations where women stopped using contraception due to a desire for pregnancy were treated as “Competing Events” rather than standard failures [22].

#### 2.3.4. Independent Variables

Predictors were selected based on a theory‐driven framework and included sociodemographic factors (age, education, religion, wealth, occupation, and residence) and information access proxies (mobile phone ownership, internet use, and media exposure).

### 2.4. Statistical Analysis

All statistical analyses were performed using Stata Version 16.1. Because traditional survival models (such as the standard Cox proportional hazards or simple parametric models) can yield biased estimates when competing events exist, the Fine–Gray subdistribution hazards model (stcrreg) was implemented as the primary analytical framework. This model treats the “desire for pregnancy” as a competing risk rather than a standard right‐censored observation, allowing for the unbiased estimation of adjusted subdistribution hazard ratios (aSHR). This ensures that intentional fertility exits are statistically accounted for as events that prevent the occurrence of the primary “in‐need” discontinuation outcome.

#### 2.4.1. Bivariate Analysis

A logistic descriptive comparison was initially conducted to assess the baseline association between covariates and the odds of discontinuation. Unlike a simple chi‐square test, this approach provided crude odds ratios (COR) and standard errors (SE), describing the magnitude and direction of associations while strictly respecting the survey′s complex sampling design.

#### 2.4.2. Competing Risks Framework

To accurately isolate “in‐need” discontinuation, the Fine–Gray subdistribution hazard model shares a semiparametric structure focused directly on the cumulative incidence function (CIF). This mathematical framework ensures that individuals who experience the competing event (intentional pregnancy) remain in the risk set with appropriate weighting, preventing the upward bias inherent in standard survival functions.

#### 2.4.3. Reproducibility and Policy Benchmarks

The adjusted cumulative incidence of “in‐need” contraceptive discontinuation was calculated at the 12‐month interval to provide actionable policy benchmarks for contraceptive persistence. To ensure the transparency and total reproducibility of these results, the complete Stata replication workflow (do‐file) including data recoding, survey configurations, and competing risks syntax is provided as Supporting Information 1.

#### 2.4.4. Model Diagnostics

Multicollinearity among predictors was rigorously assessed using variance inflation factors (VIF). A mean VIF of 1.40 (with all individual VIFs < 2.1) confirmed that the independence of covariates was maintained.

## 3. Results

### 3.1. Bivariate Logistic Regression Analysis of Factors Associated With Contraceptive Discontinuation

The bivariate logistic regression analysis (Table 1) revealed that most sociodemographic and reproductive factors did not have a statistically significant crude association with the odds of contraceptive discontinuation at the 5% significance level. However, two variables, occupational group and mobile phone ownership, emerged as significant predictors in this baseline model.

**Table 1:** Bivariate logistic regression of factors associated with contraceptive discontinuation (*n* = 2318).

Variable	Category	Crude odds ratio	Std. error	95% conf. interval	*p*
Age group	15–24	1.00 (Ref)	—	—	—
	25–34	1.18	0.43	(0.57–2.42)	0.653
	35–49	0.69	0.26	(0.33–1.45)	0.324

Education	No education	1.00 (Ref)	—	—	—
	Primary	1.09	0.35	(0.58–2.05)	0.787
	Secondary	1.23	0.63	(0.45–3.35)	0.685
	Higher	1.78	1.21	(0.47–6.74)	0.396

Religion	Orthodox/Catholic	1.00 (Ref)	—	—	—
	Protestant	0.70	0.25	(0.35–1.40)	0.314
	Muslim	1.30	0.47	(0.64–2.64)	0.463

Occupation	Unemployed	1.00 (Ref)	—	—	—
	Prof/formal	1.17	1.00	(0.22–6.24)	0.853
	Sales/service	0.52	0.16	(0.28–0.96)	0.036 ^∗^
	Manual/other	0.57	0.28	(0.22–1.49)	0.251

Own mobile	No	1.00 (Ref)	—	—	—
	Yes	1.94	0.61	(1.04–3.61)	0.037 ^∗^

Wealth index	Richest	1.00 (Ref)	—	—	—
	Poorest	2.47	1.49	(0.75–8.09)	0.135
	Poorer	0.63	0.31	(0.23–1.68)	0.352
	Middle	1.14	0.61	(0.40–3.26)	0.809
	Richer	1.01	0.51	(0.38–2.70)	0.981

Residence	Urban	1.00 (Ref)	—	—	—
	Rural	0.91	0.30	(0.48–1.73)	0.770

Media exposure					
Radio	Never	1.00 (Ref)	—	—	—
	< Once/week	0.91	0.33	(0.44–1.84)	0.784
	At least once	0.64	0.21	(0.34–1.22)	0.178

TV	Never	1.00 (Ref)	—	—	—
	< Once/week	0.77	0.31	(0.35–1.71)	0.521
	At least once	1.23	0.45	(0.61–2.51)	0.564

FP info at HF	No	1.00 (Ref)	—	—	—
	Yes	1.18	0.42	(0.59–2.36)	0.634

Internet use	No	1.00 (Ref)	—	—	—
	Yes	0.87	0.59	0.22–3.31	0.834

Fertility preference	Want to have another	1.00 (Ref)	—	—	—
	Undecided	1.13	0.63	0.37–3.40	0.834
	No more	0.91	0.24	0.54–1.54	0.716

Parity	None	1.00 (Ref)	—	—	—
	1–2	0.85	0.48	0.28–2.57	0.779
	3–4	0.96	0.48	0.36–2.55	0.936
	≥ 5	0.52	0.26	0.20–1.40	0.196

Abbreviations: FP, family planning; HF, health facility; Ref, reference.

^∗^ *p* ≤ 0.05.

Women working in sales and service occupations had 48% lower odds of discontinuation compared with unemployed women (COR = 0.52; 95% CI: 0.28–0.96; *p* = 0.036). Conversely, women who owned a mobile phone exhibited significantly higher odds of stopping their contraceptive method, showing a nearly twofold increase in the crude odds of discontinuation (COR = 1.94; 95% CI: 1.04–3.61; *p* = 0.037) compared with nonowners.

Other key variables, including age group, education level, and residence, showed no significant crude impact on the likelihood of discontinuation (*p* > 0.05). Specifically, although women with higher education (COR = 1.78) and those in the poorest wealth quintile (COR = 2.47) showed higher point estimates for the odds of stopping, these results were characterized by wide confidence intervals and did not reach statistical significance. Similarly, behavioral and access factors, such as receiving family planning information at a health facility (*p* = 0.634), media exposure through radio or television, and internet usage (*p* = 0.834), were not significantly associated with the outcome in this binary framework. Furthermore, reproductive history markers including fertility preference and parity did not demonstrate a significant crude relationship with discontinuation. These nonsignificant bivariate findings provide a baseline justification for the subsequent transition to a multivariable survival analysis, which accounts for the temporal risk and duration of use.

### 3.2. Descriptive Analysis of Contraceptive Discontinuation

For the descriptive results in Table 2, we calculated method‐specific discontinuation rates using row percentages, defines the probability of an event (in‐need vs. competing) within each specific method cohort. To determine if the differences in discontinuation reasons across these methods were statistically significant, we used a survey‐weighted Pearson′s chi‐square test (Rao–Scott correction). This test accounts for the complex sampling frame of the 2016 EDHS.

**Table 2:** Weighted modern contraceptive discontinuation rates by method and reason for stopping (EDHS 2016).

Contraceptive method	Discontinued “in‐need” (%)	Competing event (%)	Still using/censored (%)	Total episodes (*N*)
Pill	37.5%	60.0%	2.5%	280
IUD	36.6%	63.4%	0.0%	41
Injections	**29.1%**	66.9%	4.0%	1596
Implant/norplant	34.5%	60.7%	4.8%	310
Traditional	27.0%	71.4%	1.6%	63
Others	32.1%	64.3%	3.6%	28
Total sample	**31.0%**	**65.2%**	**3.8%**	**2318**

*Note:* *p* = 0.057 (Design‐based Pearson′s chi‐square test). This represents the bivariate association between contraceptive method type and the reason for discontinuation. Bold text is used to highlight the overall total sample rates and summary findings.

The overall analysis of 2318 contraceptive episodes reveals a nuanced transition in reproductive behavior among married women in Ethiopia. Of all initiated episodes, only a small fraction (3.8%) remained in continuous use at the time of the survey (censored), whereas the majority resulted in a discontinuation event. A critical distinction emerges when decomposing the reasons for discontinuation into “In‐Need” cessation and “Competing Reproductive Events.” The results indicate that the overall weighted 12‐month rate of discontinuation, whereas “In‐Need” (defined as stopping due to side effects, method failure, or health concerns) was 31.0%. In contrast, a significantly larger proportion of episodes (65.2%) were discontinued due to competing events, primarily the intentional desire to become pregnant or the decision to switch to a different contraceptive method.

Method‐specific analysis further highlights variations in contraceptive persistence across different technologies. Contrary to traditional assumptions, the highest probability of “In‐Need” discontinuation was observed among women using the pill (37.5%), followed by IUD users (36.6%), and implant users (34.5%). Conversely, injectable users, who constitute the largest segment of the study population, exhibited a comparatively lower rate of “In‐Need” discontinuation at 29.1%. These descriptive findings suggest that although short‐acting oral methods face higher attrition due to health barriers, long‐acting methods and injectables demonstrate relatively higher levels of continuity within the Ethiopian healthcare context. Furthermore, the high prevalence of discontinuation for planned pregnancy underscores that a substantial portion of contraceptive cessation reflects successful fertility regulation rather than a failure of family planning service delivery.

### 3.3. Factors Associated With “In‐Need” Contraceptive Discontinuation

To identify the sociodemographic predictors of “In‐Need” discontinuation, a multivariable Fine–Gray competing risks regression model was executed (Table 3), treating intentional pregnancy as a competing risk.

**Table 3:** Factors associated with contraceptive discontinuation “In‐Need”: Results from the Fine–Gray subdistribution hazard competing‐risks regression model.

Variable	Category	Crude SHR (95% CI)	Adjusted SHR (95% CI)	*p* (Adj)
Age group	15–24 (Ref)	1.00	1.00	—
	25–34	1.15 (0.82–1.61)	1.08 (0.75–1.56)	0.679
	35–49	**1.94 (1.40–2.71)**	**1.85 (1.26–2.71)**	**0.002**

Education	No education (Ref)	1.00	1.00	—
	Primary	0.85 (0.65–1.11)	0.98 (0.74–1.29)	0.862
	Secondary	**0.57 (0.39–0.84)**	**0.62 (0.40–0.96)**	**0.033**
	Higher	0.67 (0.38–1.19)	1.00 (0.49–2.07)	0.991

Religion	Orthodox (Ref)	1.00	1.00	—
	Catholic	**4.47 (1.97–10.14)**	**4.65 (2.06–10.49)**	**< 0.001**
	Protestant	1.05 (0.78–1.41)	0.99 (0.74–1.32)	0.940
	Muslim	0.92 (0.65–1.32)	0.91 (0.64–1.29)	0.597

Occupation	Not working (Ref)	1.00	1.00	—
	Prof/formal	0.71 (0.34–1.47)	0.69 (0.28–1.67)	0.411
	Sales/service	1.08 (0.83–1.40)	1.06 (0.82–1.37)	0.670
	Manual/other	1.38 (0.96–1.99)	**1.56 (1.08–2.25)**	**0.018**

Fertility pref	No more (Ref)	1.00	1.00	—
	Want another	**0.69 (0.54–0.87)**	0.85 (0.65–1.11)	0.244
	Undecided	**0.55 (0.33–0.94)**	**0.55 (0.32–0.93)**	**0.026**

*Note:* Bold values indicate independent variable categories that are statistically significant at the *p*<0.05 level in the multivariable Fine–Gray subdistribution hazards model.

Abbreviations: aSHR, adjusted subdistribution hazard ratio; CI, confidence interval; Ref, reference.

### 3.4. Sociodemographic Determinants

The risk of discontinuing a method while still in need was significantly higher among older women. Those in the 35–49 age bracket experienced an 85% higher subdistribution hazard of discontinuation (aSHR: 1.85; 95% CI: 1.26–2.71) compared with women aged 15–24.

Regarding educational attainment, only secondary education reached statistical significance as a protective factor; women with a secondary education were 38% less likely to discontinue their method compared with those with no formal schooling (aSHR: 0.62; 95% CI: 0.40–0.96). Notably, having only a primary education (aSHR: 0.98; 95% CI: 0.74–1.29) or a higher education (aSHR: 1.00; 95% CI: 0.49–2.07) did not significantly alter the hazard of discontinuation in the adjusted model.

Religious affiliation also emerged as a strong predictor. Catholic women faced a significantly higher hazard of “In‐Need” discontinuation compared with Orthodox women (aSHR: 4.65; 95% CI: 2.06–10.49), whereas no significant differences were observed for Protestant or Muslim users.

**Figure 1:**
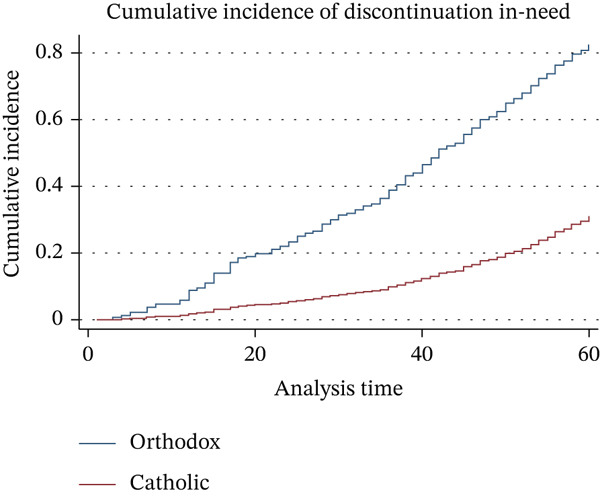
Cumulative incidence function of contraceptive discontinuation “In‐Need” among Ethiopian women, stratified by religious affiliation (EDHS 2016).

The CIF stratified by religion (Figure 1) highlights this distinct behavioral pattern.

**Figure 2:**
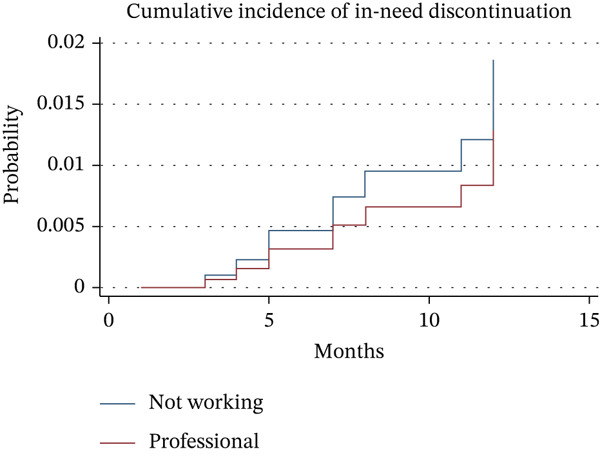
Cumulative incidence function of in‐need discontinuation among Ethiopian women, stratified by occupational group (EDHS 2016).

In terms of occupation, women engaged in manual or other labor categories had a 56% increased subdistribution hazard of discontinuation (aSHR: 1.56; 95% CI: 1.08–2.25) relative to those who were not working. This relationship is plotted via the cumulative incidence estimates over time in (Figure 2).

### 3.5. Reproductive and Contextual Factors

Fertility preference significantly influenced contraceptive persistence. Women who reported being “undecided” about their future fertility were 45% less likely to discontinue their method (aSHR: 0.55; 95% CI: 0.32–0.93) than those who expressed a definitive desire for no more children. Interestingly, variables representing economic status (wealth index), geographic location (residence), and information access (mobile phone ownership and internet use) did not show statistically significant associations with the hazard of “In‐Need” discontinuation in the final multivariable competing risks framework (*p* > 0.05) (Table 4).

**Table 4:** Multivariable Fine–Gray competing risks regression of factors associated with “In‐Need” contraceptive discontinuation.

Variable	Category	aSHR	95% conf. interval	*p*
Age group	15–24	1.00	(Reference)	—
	25–34	1.08	0.75–1.56	0.679
	**35–49**	**1.85**	**1.26–2.71**	**0.002**

Occupation	Not working	1.00	(Reference)	—
	Prof/formal	0.69	0.28–1.67	0.411
	Sales/service	1.06	0.81–1.37	0.670
	**Manual/other**	**1.56**	**1.08–2.25**	**0.018**

Education	No education	1.00	(Reference)	—
	Primary	0.98	0.74–1.29	0.862
	**Secondary**	**0.62**	**0.40–0.96**	**0.033**
	Higher	1.00	0.49–2.07	0.991

Religion	Orthodox	1.00	(Reference)	—
	**Catholic**	**4.65**	**2.06–10.49**	**<0.001**
	Protestant	0.99	0.74–1.32	0.940
	Muslim	0.91	0.64–1.29	0.597

Fertility preference	No more (Ref)	1.00	(Reference)	—
	Want another	0.85	0.65–1.11	0.244
	**Undecided**	**0.55**	**0.32–0.93**	**0.026**

*Note:* SHR > 1 indicates increased hazard of in‐need discontinuation. Model adjusted for all listed variables and accounts for clustering at the PSU level. Bold values indicate independent variable categories that are statistically significant at the *p*<0.05 level in the multivariable Fine–Gray subdistribution hazards model.

Abbreviation: aSHR, adjusted subdistribution hazard ratio.

### 3.6. Overall Incidence and Vulnerability Profile

The adjusted 12‐month cumulative incidence of “in‐need” contraceptive discontinuation varied significantly across sociodemographic strata (Table 5), confirming that risk is concentrated within specific vulnerable cohorts.

**Table 5:** Adjusted 12‐month cumulative incidence of “In‐Need” contraceptive discontinuation among women in Ethiopia (EDHS 2016).

Variable	Category	Adjusted 12‐month CIF (%)	95% confidence interval
Age group	15–24 years (Ref)	**1.58**	(1.18–2.12)
	35–49 years	**2.91**	(2.15–3.94)
Education level	No education (Ref)	**1.85**	(1.37–2.51)
	Secondary	**1.27**	(0.84–1.92)
Occupation	Prof./formal (Ref)	**1.29**	(0.81–2.05)
	Manual/other	**1.97**	(1.45–2.68)
Fertility preference	Want have another (Ref)	**1.89**	(1.40–2.54)
	Undecided	**1.03**	(0.61–1.75)
Religion	Orthodox (Ref)	**1.85**	(1.37–2.51)
	Catholic	**1.95**	(0.92–4.12)

*Note:* Estimates are adjusted for all other variables in the table using a competing risks Gompertz model, accounting for “not‐in‐need” reasons (desire for pregnancy) as a competing event. Bold text is used to denote the main sociodemographic variable headers and their respective reference categories (Ref) evaluated for the 12‐month cumulative incidence.

Abbreviations: CIF, cumulative incidence function; Ref, reference category.

#### 3.6.1. Age and Education Drivers

A significant positive risk trend with age was observed; women in the oldest reproductive age cohort (35–49 years) exhibited the highest probability of discontinuation at 2.91%, nearly double the risk of the youngest cohort (1.58%). Education remained a robust protective factor, as women with a secondary education demonstrated a reduced 12‐month incidence of 1.27% compared with 1.85% among those with no formal schooling.

#### 3.6.2. Economic and Cultural Determinants

Occupational demands significantly influenced persistence. Women engaged in manual labor faced a higher incidence of 1.97%, whereas those in professional or formal roles showed a lower probability of 1.29%. This suggests that method‐related side effects may impose a higher functional and economic burden on women in physically demanding jobs. Furthermore, although the absolute number of Catholic women was small, their specific risk intensity remained elevated at 1.95%.

#### 3.6.3. The Role of Fertility Ambivalence

One of the most nuanced findings involves fertility intentionality. Women who were “undecided” about their future fertility goals demonstrated the highest level of persistence, with a 12‐month discontinuation probability of only 1.03%. This challenges the traditional assumption that only those with firm limiting goals maintain consistent use, suggesting instead that fertility ambivalence may foster a more cautious and stable approach to contraceptive maintenance (Table 5).

## 4. Discussion

This study is the first to utilize a competing risks framework to estimate the adjusted cumulative incidence of “in‐need” contraceptive discontinuation in Ethiopia. Our findings reveal a complex profile of contraceptive vulnerability across Ethiopia. The “in‐need” discontinuation is not uniform; it is concentrated among older women and those with lower educational attainment nationally. The significant protective effect of fertility “undecidedness” challenges the traditional view that only those with firm limiting goals are consistent users. These results suggest that national family planning programs in Ethiopia should tailor interventions specifically toward older, less‐educated women who are at the highest statistical risk of stopping method use despite a continued need for pregnancy prevention.

### 4.1. The Dynamics of Fertility Preference and Intentionality

A major contribution of this study is the nuanced finding regarding the role of fertility preferences in contraceptive persistence. After adjusting for potential confounders and accounting for the desire for pregnancy as a competing risk, women who wanted another child did not exhibit a statistically significant difference in their hazard of “in‐need” discontinuation compared with those who wanted no more children (aSHR: 0.85; 95% CI: 0.65–1.11).

This finding aligns with recent evidence from India and previous longitudinal assessments in Ethiopia, which suggest that when the analysis is restricted to method‐related barriers such as side effects, health concerns, or opposition, the underlying reproductive intention becomes a secondary factor [22–26]. In this context, clinical side effects may act as a “biological equalizer,” exerting a universal pressure on users regardless of whether their ultimate goal is to space or limit births.

However, this result stands in contrast to studies from other regions, such as Kenya and the Philippines, where “limiters” (women wanting no more children) typically show significantly higher persistence than “spacers” [25, 26].

In those settings, it is argued that women with a temporary need for protection have a lower “threshold of tolerance” for side effects compared with those who have completed their families. The lack of such a distinction in our Ethiopian sample may indicate that the perceived socioeconomic and health risks of an unintended pregnancy are equally high for both groups, leading to a similar level of motivation to persist with contraception despite method‐related challenges.

Interestingly, our study did find that women who were undecided about their future fertility had a significantly lower hazard of discontinuation (aSHR: 0.55; 95% CI: 0.32–0.93). This suggests that ambivalence toward future childbearing may lead to a more cautious approach to contraceptive use, perhaps reflecting a strong desire to avoid unplanned pregnancy until a firm reproductive decision is reached.

### 4.2. Age and Cumulative Risk

The study identified an elevated subdistribution risk of in‐need discontinuation among older cohorts, with women in the oldest age group (35–49 years) being nearly twice as likely to discontinue compared with those aged 15–24 (aSHR: 1.85; 95% CI: 1.26–2.71). This finding is particularly striking as it contradicts a large body of literature from other sub‐Saharan African contexts, which typically identifies younger age as the primary risk factor for method cessation.

[10, 18, 27, 28]. In those settings, younger users are often viewed as more vulnerable due to less experience with methods and higher rates of “method‐switching.”

However, our results align with emerging evidence from Ethiopia suggesting that older users face a distinct set of pressures [29–31]. The increased hazard in later reproductive years may be attributed to a higher cumulative experience of side effects over time. Furthermore, a “perceived decline in fecundity” among women approaching menopause may lead to a lower perceived risk of conception, making them more likely to prematurely stop using modern methods when faced with minor side effects or health concerns.

This highlights a critical gap in family planning programs, which often prioritize adolescents and young women. Our findings suggest that older users require specific clinical counseling that addresses their unique health concerns and clarifies the continued risk of pregnancy until the permanent cessation of menstruation. Shifting the programmatic focus to include these long‐term users is essential for reducing the unmet need among women who have already achieved or are near their desired family size.

### 4.3. Socioeconomic and Occupational Barriers

The protective effect of education was evident in this study, particularly for women with secondary education, who were 38% less likely to discontinue compared with those with no formal schooling (aSHR: 0.62; 95% CI: 0.40–0.96). This aligns with previous national evidence from Ethiopia and sub‐Saharan Africa, which suggests that higher educational attainment enhances health literacy and empowers women to navigate method‐related expectations [13, 24, 30–33]. Educated women may be better equipped to interpret clinical information regarding side effects, reducing the likelihood of “panic discontinuation” and facilitating a transition to alternative methods when initial choices prove unsatisfactory. This contrasts with findings from Nigeria, where higher education has occasionally been linked to higher discontinuation due to increased sensitivity to side effects that conflict with professional responsibilities [34, 35].

The multivariable Fine–Gray analysis showed that women in manual occupations had a 56% higher subdistribution hazard of in‐need discontinuation compared with those who were not working (aSHR: 1.56; 95% CI: 1.08–2.25; *p* = 0.018).

This finding is consistent with research in Bangladesh and Ethiopia, which indicates that physically demanding occupations exacerbate the perceived burden of side effects like dizziness or fatigue, which directly impact daily labor productivity [24]. Furthermore, women in these sectors often face “time‐poverty,” creating logistical barriers to accessing follow‐up care for side‐effect management.

However, our results diverge from findings in Ghana, where manual laborers showed higher persistence driven by the economic necessity of avoiding the workplace disruptions associated with a new pregnancy [36]. In the Ethiopian context, it appears that the immediate physical and logistical strain of manual labor outweighs the long‐term fear of pregnancy‐related work disruption. These results underscore the need for family planning services to provide more flexible and community‐based follow‐up care that reaches women in their workplaces or homes, reducing the “access cost” for the most economically active yet vulnerable users.

Interestingly, the wealth index and place of residence were nonsignificant in the multivariable model. This suggests that once a method is initiated, clinical side effects act as a “biological equalizer” that transcends economic and geographic boundaries. Furthermore, the null finding for mobile phone ownership and internet use provides a critical policy insight. It signals that digital access is not a substitute for high‐quality face‐to‐face clinical counseling.

### 4.4. Religious Influence and Cultural Context

The analysis revealed a striking and statistically robust association between religious affiliation and contraceptive persistence. Catholic women exhibited a dramatically higher hazard of in‐need discontinuation (aSHR: 4.65; 95% CI: 2.06–10.49) compared with the Orthodox majority. This finding aligns with previous evidence from Ethiopia and other sub‐Saharan African contexts, which highlights the profound influence of religious doctrine on reproductive behavior [36–38]. In specific religious communities, the pressure to discontinue may stem from deeply rooted doctrinal opposition to modern “artificial” methods, often leading to method cessation even when a woman expresses a clear desire to delay childbearing. In these cases, the “in‐need” discontinuation is likely driven by internal moral conflict or external social pressure from the community and religious leaders.

However, our results contrast with findings from Latin America and parts of West Africa, where the “Catholic effect” has been found to be negligible or secondary to secular socioeconomic factors [39, 40]. In those regions, personal reproductive goals often take precedence over official church teachings. The unique intensity of the association in our Ethiopian sample may reflect the localized influence of religious institutions in providing or influencing health norms.

This suggests that in Ethiopia, religious affiliation is not merely a demographic marker but a powerful contextual determinant of contraceptive stability. This finding points to a critical need for family planning programs to move beyond purely clinical models. Effective interventions must engage directly with religious leaders to bridge the gap between doctrinal values and the reproductive health needs of their congregants. By fostering a dialogue that aligns family planning with broader values of maternal health and family well‐being, programs may reduce the “religious hazard” and support women in achieving their desired reproductive outcomes within their cultural and spiritual frameworks.

Finally, these nonsignificant socioeconomic indicators should be interpreted as contextual proxies rather than direct causal predictors. Since variables like wealth and ICT ownership were measured at the time of the interview, they reflect the woman′s current environment rather than her exact status at the start of the contraceptive episode.

## 5. Strengths and Limitations

### 5.1. Strengths

A primary strength of this analysis is the application of the Fine–Gray subdistribution hazards model, which represents a significant methodological advancement over traditional survival analyses. By accounting for the desire for pregnancy as a competing event, this approach avoids the overestimation of “in‐need” discontinuation risks, thereby providing more accurate and actionable parameters for public health planning and family planning quality improvement.

Furthermore, the use of nationally representative data from the EDHS ensures that the findings are generalizable across diverse regional and sociodemographic strata. Additionally, the inclusion of a comprehensive array of sociodemographic and method‐related predictors provides a holistic view of the systemic barriers to contraceptive security in Ethiopia.

### 5.2. Limitations

The study relies on retrospective data from the EDHS contraceptive calendar, which is subject to recall bias over a 60‐month period. Because the study is based on a cross‐sectional survey, key sociodemographic variables such as education and occupation were treated as time‐fixed, which may not capture transitions during the 5‐year observation window. Furthermore, influential factors such as the quality of provider‐client counseling, specific types of side effects, and male partner involvement could not be evaluated due to dataset limitations.

Additionally, women who stopped a method to immediately start another were classified as discontinued “in‐need” if the reason was related to side effects, capturing method‐specific attrition rather than total contraceptive cessation. Finally, although Catholic affiliation was associated with a significantly higher hazard, the small number of Catholic women in the survey (*N* = 17) resulted in wide confidence intervals; thus, these results must be interpreted as indicative rather than definitive for this specific subgroup.

## 6. Conclusion

“In‐need” contraceptive discontinuation in Ethiopia is driven by a complex interplay of age, educational attainment, and occupational physical demands. The finding that older and less‐educated women are at higher risk suggests that national family planning programs should move beyond adolescent‐focused strategies to include targeted clinical counseling for long‐term users. Furthermore, the economic impact of side effects on manual laborers necessitates more flexible, community‐based follow‐up care to ensure contraceptive security for Ethiopia′s most economically vulnerable women.

**Nomenclature**
aSHR: adjusted subdistribution hazard ratio
CIF: cumulative incidence function
Crude SHR: crude subdistribution hazard ratio
DF: degrees of freedom
EDHS: Ethiopia demographic and health survey
FP: family planning
GDP: gross domestic product
HF: health facility
IUD: intrauterine device
PSU: primary sampling unit
SDGs: sustainable development goals
SE: standard errors
VIF: variance inflation factors

## Author Contributions

A.A.B. and A.A.D. conceptualized and designed the study, performed the formal data analysis and interpretation, and prepared the initial draft of the manuscript. A.A.B., A.A.D., D.M., A.G.W., N.M.B., G.A.B., H.K., S.A.T., and B.A. were involved in critically commenting on and revising the manuscript for intellectual content, providing final approval of the version to be published.

## Funding

No funding was received for this manuscript.

## Disclosure

All authors read and approved the final manuscript.

## Ethics Statement

The analysis conducted for this study utilized secondary data sourced from the 2016 EDHS. Formal authorization to access and analyze the publicly available, anonymized dataset was obtained from the DHS Program and the Central Statistical Agency (CSA) of Ethiopia. Because the data were secondary and entirely anonymous, the study adhered to standard ethical protocols. The original DHS data collection process ensured that informed consent was obtained from all respondents, and all procedures complied with international ethical guidelines regarding participant confidentiality and anonymity.

## Consent

The authors have nothing to report.

## Conflicts of Interest

The authors declare no conflicts of interest.

## Supporting information


**Supporting Information** Additional supporting information can be found online in the Supporting Information section. Supporting Information 1 (.do file): Stata replication workflow. This file contains the complete Stata commands used for data recoding, the Fine–Gray competing risks, and the generation of cumulative incidence functions.

## Data Availability

The datasets analyzed during the current study were derived from the 2016 Ethiopia Demographic and Health Survey (EDHS), which is publicly available upon request and authorization. The data are maintained and distributed by the DHS Program (Measure DHS). Access to the datasets used and/or analyzed during the current study can be obtained directly from the DHS Program′s online repository by creating an account and submitting a simple data use request. The datasets are available at the following official link: https://www.dhsprogram.com/data/available-datasets.cfm. Alternatively, the specific data can be made available from the corresponding author upon reasonable request.
