# Supplementary material for: Duration and Predictors of “In‐Need” Contraceptive Discontinuation in Ethiopia: A Competing Risks Analysis Using the Fine–Gray Subdistribution Hazards Model
Source: Biomed Res Int. 2026 Jun 16;2026:9045082. doi: 10.1155/bmri/9045082 (PMC13270355; doi:10.1155/bmri/9045082)
Supplement: Supplementary file 1 — Supporting Information Additional supporting information can be found online in the Supporting Information section. Supporting Information 1 (.do file): Stata replication workflow. This file contains the complete Stata commands used for data recoding, the Fine–Gray competing risks, and the generation of cumulative incidence functions. [file BMRI-2026-9045082-s001.docx]

* PROJECT: Duration and Predictors of 'In-Need' Contraceptive Discontinuation

* DATA: Ethiopia Demographic and Health Survey (EDHS) 2016

* MODEL: Competing Risks Gompertz Proportional Hazards

********************************************************************************

* PREPARATION: Set working directory and memory

clear all

set more off

/* STEP 1: DATA CLEANING & RECODING (MANUSCRIPT SECTION: METHODS) */

* 1.1 Define the survey design for Ethiopia 2016

svyset v001 [pweight=v005], strata(v023) vce(linearized)

* 1.2 Recode the Outcome: Reasons for Discontinuation

* Outcome: 0=Censored, 1=In-Need (Side effects/Health), 2=Competing (Pregnancy)

gen event = 0

replace event = 1 if vcase == 1 | vcase == 2 | vcase == 3 // Side effects, Health, Opposition

replace event = 2 if vcase == 4 // Wanted pregnancy (Competing risk)

label define event_lab 0 "Censored" 1 "In-Need" 2 "Want Pregnancy"

label values event event_lab

* 1.3 Recode Covariates (Table 1 & Table 5 Variables)

* Age Group

recode v012 (15/24=0 "15-24") (25/34=1 "25-34") (35/49=2 "35-49"), gen(age_gp)

* Education Level

recode v106 (0=0 "No Education") (1=1 "Primary") (2=2 "Secondary") (3=3 "Higher"), gen(Education_level)

tab age_gp

* Occupation Group

gen occ_group = .

replace occ_group = 1 if v717 == 0 // Not working

replace occ_group = 2 if v717 == 1 | v717 == 2 // Prof/Formal

replace occ_group = 3 if v717 == 3 | v717 == 5 // Sales & Service

replace occ_group = 4 if v717 == 4 // Agriculture

replace occ_group = 5 if v717 >= 6 // Manual/Other

label define occ_lab 1 "Not working" 2 "Prof/Formal" 3 "Sales" 4 "Agri" 5 "Manual"

label values occ_group occ_lab

* Fertility Preference

gen Fertil_preference = .

replace Fertil_preference = 1 if v605 == 1 | v605 == 2 // Want more

replace Fertil_preference = 2 if v605 == 5 // Undecided

replace Fertil_preference = 3 if v605 == 4 | v605 == 6 // Limit/Sterilized

gen religion = .

replace religion = 1 if religion_1 ==1

replace religion = 2 if religion_1 ==2

replace religion = 3 if religion_1 ==3

replace religion = 4 if religion_1 ==4

* Define and attach the value labels

label define religion 1 "Orthodox" 2 "Catholic" 3 "Protestant" 4 "Muslim"

label values religion religion

tab religion

gen wealth_index = .

replace wealth_index = 1 if v190==0

replace wealth_index = 2 if v190==1

replace wealth_index = 3 if v190==2

replace wealth_index = 4 if v190==3

replace wealth_index = 5 if v190==4

* Define and attach the value labels

label define wealth_index 1"poorest" 2 "poorer" 3 " middle" 4 "richer"5"richest"

label values wealth_index wealth_index

tab wealth_index

gen residence = .

replace residence = 1 if v025==1

replace residence = 2 if v025==2

*Define and attach the value labels

label define residence 1"urban " 2 "rural"

label values residence residence

tab residence

gen Mobile_own= .

replace Mobile_own = 1 if v169a==0

replace Mobile_own = 2 if v169a==1

*Define and attach the value labels

label define Mobile_own 1"no" 2 "yes"

label values Mobile_own Mobile_own

tab Mobile_own

gen internet_use = .

replace internet_use = 1 if Use_internet==0

replace internet_use = 2 if Use_internet==1

*Define and attach the value labels

label define internet_use 1"never" 2 "yes_last_12months"

label values internet_use internet_use

tab internet_use

gen parity = .

replace parity = 1 if No_living_ch ==0

replace parity = 2 if No_living_ch ==1

replace parity = 3 if No_living_ch ==2

replace parity = 4 if No_living_ch ==3

* Define and attach the value labels

label define parity 1 "none " 2 "1-2" 3 "3-4" 4 ">=5"

label values parity parity

tab parity

gen told_about_FP = .

replace told_about_FP = 1 if v395==0

replace told_about_FP = 2 if v395==1

*Define and attach the value labels

label define told_about_FP 1"no" 2 "yes"

label values told_about_FP told_about_FP

tab told_about_FP

gen freq_listen_radio = .

replace freq_listen_radio = 1 if v158 ==0

replace freq_listen_radio = 2 if v158==1

replace freq_listen_radio = 3 if v158 ==2

* Define and attach the value labels

label define freq_listen_radio 1 "not at all" 2 "less than once a week " 3 "at least once a week"

label values freq_listen_radio freq_listen_radio

tab freq_listen_radio

gen freq_watching_TV = .

replace freq_watching_TV= 1 if v159==0

replace freq_watching_TV = 2 if v159==1

replace freq_watching_TV = 3 if v159 ==2

* Define and attach the value labels

label define freq_watching_TV 1 "not at all" 2 "less than once a week " 3 "at least once a week"

label values freq_watching_TV freq_watching_TV

tab freq_watching_TV

/* STEP 2: DESCRIPTIVE & BIVARIATE ANALYSIS (MANUSCRIPT SECTION: TABLE 1 & 2) */

* 2.1 Table 1: Bivariate Logistic Regression (Crude Odds Ratios)

foreach var of varlist age_gp Education_level occ_group Religion {

svy: logistic event_binary i.`var' // where event_binary is 1 if event==1

}

lookfor event

lookfor discont

rename _d event_binary

foreach var of varlist age_gp Education_level occ_group Religion {

svy: logistic event_binary i.`var'

}

* --- 1. Age Group (Reference: 15-24) ---

svy: logistic event i.age_gp

* --- 2. Education (Reference: No Education / 0) ---

svy: logistic event i.Education_level

* --- 3. Religion (Reference: Orthodox/ 1) ---

svy: logistic event i.religion

* --- 4. Occupation (Reference: Unemployed / 0) ---

svy: logistic event i.occ_group

* --- 5. Mobile_own (Reference: No / 0) ---

svy: logistic event i.Mobile_own

* --- 6. Wealth Index (Reference: Richest / 5) ---

* If Richest is coded as 5 in your data, set ib5.

svy: logistic event ib5.wealth_index

* --- 7. Residence (Reference: Urban / 1) ---

svy: logistic event ib1.residence

* --- 8. Media Exposure: freq_listen_radio (Reference: Never / 0) ---

svy: logistic event i.freq_listen_radio

* --- 9. Media Exposure: freq_watching_TV(Reference: Never / 0) ---

svy: logistic event i.freq_watching_TV

* --- 10. told_about_FP (Reference: No / 0) ---

svy: logistic event i.told_about_FP

* --- 11. Internet Use (Reference: No / 0) ---

svy: logistic event i.internet_use

* --- 12. Fertil_preference (Reference: Want to have another / 1) ---

svy: logistic event i.Fertil_preference

* --- 13. Parity (Reference: None / 0) ---

svy: logistic event i.parity

=============================

*Table 2

* 1. Create the standardized weight variable from the EDHS weight (v005)

gen weight = v005 / 1000000

* 2. Declare the complex survey design (using your standardized weights)

svyset v021 [pweight=weight], strata(v022) vce(linearized)

* 3. Check your existing Method variable categories

numlabel, add

tab last_Meth_discontinuedM // Assumes your method variable includes Pill, IUD, Injections, Implant, etc.

* 3. Make sure your Event/Reason status variable is coded precisely into 3 categories:

* 0 = Still Using / Censored

* 1 = Discontinued "In-Need"

* 2 = Competing Event (e.g., Intended Pregnancy)

* 1. Re-verify that your survey weights and design matrix are active

capture gen weight = v005 / 1000000

svyset v021 [pweight=weight], strata(v022) vce(linearized)

* 2. Calculate the cross-tabulation using your precise layout options

svy: tab last_Meth_discontinuedM event, row percent format(%9.1f) count

* 4. Produce the raw absolute sample frequencies (N) to fill your column totals

tab last_Meth_discontinuedM, missing

tab event

=========================

drop if current_contraceptive_Method == 0

* Recode into a brand new clean variable 'method_6cat'

recode current_contraceptive_Method ///

(1 = 1 "Pill") ///

(2 = 2 "IUD") ///

(3 = 3 "Injections") ///

(6 = 4 "Implant/Norplant") /// <- Combines Implant and Norplant codes

(5 = 5 "Traditional") /// <- Adjust code if your traditional method has a different number

(4 7 8 9 10 = 6 "Others"), /// <- Everything else active goes into "Others"

gen(method_6cat)

/* STEP 4: MULTIVARIABLE COMPETING RISKS MODEL (MANUSCRIPT SECTION: TABLE 4) */

* 4.1 Fine-Gray Model for In-Need Discontinuation

* Compete(2) defines 'Want Pregnancy' as the competing risk

stcrreg i.age_gp i.occ_group i.Education_level i.Religion ib2.Fertil_preference, ///

compete(event==2) vce(cluster v001)

* 4.2 Proportional Hazards Assumption Check

stcoxkm, by(Education_level)

/* STEP 5: PREDICTED INCIDENCE (MANUSCRIPT SECTION: TABLE 5) */

* 5.1 Generating Cumulative Incidence Functions (CIF) at 12 months

* Example for Age Group 35-49 (category 2)

stcurve, cif at(age_gp=2) outfile(age_high, replace)

preserve

use age_high, clear

list ci1 if _t == 12

restore

* Example for Undecided Fertility Preference (category 2)

stcurve, cif at(Fertil_preference=2) outfile(fert_undecided, replace)

preserve

use fert_undecided, clear

list ci1 if _t == 12

restore
